# Magnetic exposure using Samarium Cobalt (SmC_O5_) increased proliferation and stemness of human Umbilical Cord Mesenchymal Stem Cells (hUC-MSCs)

**DOI:** 10.1038/s41598-022-12653-z

**Published:** 2022-05-26

**Authors:** Haslinda Abdul Hamid, Rajesh Ramasamy, Mohd Kamarulzaki Mustafa, Vahid Hosseinpour Sarmadi, Azizi Miskon

**Affiliations:** 1 Bio Artificial Organ and Regenerative Medicine Unit, National Defense University of Malaysia, Sungai Besi Camp, 57000 Kuala Lumpur, Malaysia; 2 Stem Cell & Immunity Research Group, Immunology Laboratory, Department of Pathology, Faculty of Medicine and Health Sciences, University Putra Malaysia, 43400 Serdang, Malaysia; 3 Department of Physics, Faculty of Applied Sciences and Technology, University Tun Hussein Onn Malaysia, Pagoh Campus, KM1, Jalan Panchor, Hub Pendidikan Tinggi Pagoh, 84600 Muar, Johor Malaysia; 4 Institutes of Regenerative Medicine, Faculty of Advanced Technologies in Medicine, Iran University of Medical Sciences, Tehran, Iran; 5 Cellular and Molecular Research Centre, Iran University of Medical Sciences, Tehran, Iran; 6 Department of Dental Radiology, Faculty of Dental Medicine, Airlangga University, Surabaya, 60132 Indonesia

**Keywords:** Biotechnology, Stem cells

## Abstract

Despite the extensive reports on the potential hazard of magnetic field (MF) exposures on humans, there are also concurrently reported on the improved proliferative property of stem cells at optimum exposure. However, the effect on mesenchymal stem cells (MSCs) remains unknown. Therefore, we aimed to investigate the impact of induced static MF (SMF) on human umbilical cord-derived mesenchymal stem cells (hUC-MSCs) using Samarium Cobalt (SmCO5). At passage 3, hUC-MSCs (1 × 10^4^) were exposed to 21.6 mT SMF by a direct exposure (DE) showed a significantly higher cell count (*p* < 0.05) in the growth kinetics assays with the shortest population doubling time relative to indirect exposure and negative control. The DE group was committed into the cell cycle with increased S phase (55.18 ± 1.38%) and G2/M phase (21.75 ± 1.38%) relative to the NC group [S-phase (13.54 ± 2.73%); G2/M phase (8.36 ± 0.28%)]. Although no significant changes were observed in the immunophenotype, the DE group showed an elevated expression of pluripotency-associated markers (*OCT4, SOX2, NANOG,* and *REX1*). These results suggest that the MFs could potentially induce proliferation of MSCs, a promising approach to promote stem cells propagation for clinical therapy and research without compromising the stemness of hUC-MSCs.

## Introduction

Recent years have witnessed a substantial breakthrough in our understanding of the human adult stem cell biology that has reflected in a surge of its therapeutic usage following improved reported clinical efficacy. From cell-based therapy^1,2^, development of bio-artificial organ^3,4^ and wound tissue repair through rapid tissue regeneration^5–7^ to the treatment of various cancer types^8,9^, the adoption of human adult stem cells through stem cell transplantation has gained a wide-spread popularity attributed to its minimal risk of host rejection and side effects despite its high therapeutic potency.

Mesenchymal stem cells (MSCs) produced in the bone marrow (BM) is considered the most common and longest utilised adult source tissues for human MSCs. Another adult sources of “adult” MSCs are umbilical cord tissues and placenta which were often discarded at birth^10^. They possess high self-renewal properties and great potential differentiation capabilities^11^. It has been shown that MSCs may give rise to cells of mesodermal lineages such as bone, adipose, cartilage, tendon, and skeletal muscle^12–14^. Several reports have been shown that MSCs also potentially differentiate into various non- mesodermal lineage tissues including pancreatic islet cells^15^, cardiac muscle^16^, hepatocyte^17^ and neural cell^18^. In comparison to other tissue-specific adult stem cells, MSCs is a preferred therapeutic agent due to the targeted homing capacity to the site of injuries and the ability to differentiate into many different mesenchymal and parenchymal cell types^19^. MSCs also possess unique reparative and immunosuppressive properties in that it secretes large amounts of pro-angiogenic, anti-inflammatory and anti-apoptotic cytokines/factors which may be responsible for the induction of tissue regeneration, transplantation tolerance and control of autoimmunity^20,21^. Attributed to the immune-privilege properties of MSCs that allows them to evade host immune response, intravascular therapy involving the intervention of MSCs remains as a low-risk clinical procedure. Further studies may be conducted to evaluate the optimal dosing and delivery method for MSCs as this factor on top of other predisposing factors involving host immune response may play a major role in steering the final clinical outcome of the therapy.

However, the therapeutically potential MSCs can be only harnessed by determining the optimal conditions required to readily stimulate their proliferation and continuous propagation for research and therapeutic purposes^22–25^. The umbilical cord tissue provides a limited source of freshly isolated cells and although studies have suggested that human umbilical cord MSCs (hUC-MSCs) exhibit a higher proliferative capacity relative to MSCs from bone marrow or adipose tissue^26,27^, there is a need for subsequent large scale in vitro expansion without modifying their stemness and differentiation capabilities. The challenge is therefore, determining an optimal condition of culture that can favour the large expansion of hUC-MSCs to meet clinical demand.

Generally, humans and other living things are naturally exposed to Earth’s MF, which is not harmful, as well as other sources of harmless MFs as obtainable from magnetized materials known as permanent magnets. The key to understanding the dichotomy between harmful and harmless forms of MFs lies in the classification of MFs based on their sources. MFs are basically of two types namely, the endogenous and exogenous fields. Endogenous fields are those MFs that are produced within the body. This type of MF occurs at various electrically excitable organs such as heart^28–30^, brain^31,32^ and eye^33^. Study also indicated MFs effected the musculoskeletal system performance^34^. On the other hand, exogenous fields are MFs produced by sources outside the body and can be classified as natural exogenous fields such as the Earth's geomagnetic field (e.g., Samarium Cobalt (SmCo_5_) or Neodymium Ferum Boron (NdFeB)) or artificial exogenous (man-made) MFs like power lines, transformers, appliances, radio transmitters, and medical devices^35,36^. Artificial exogenous MFs have been identified by many researchers and the form of MF that poses serious harmful and hazardous effects to humans and other living things on prolonged or frequent exposure. Frequent human exposure to MFs has raised serious health concerns as MF has been associated with some medical complications. Several types of cancer^37^, tumours^38^, glioblastoma^39,40^ and leukaemia^41^ have been associated with MF exposure. Since humans are faced with constant risks of being exposed to harmful forms of MFs from day-to-day activities in work places, homes, subways and other public places, researchers have focus more on exploring the harmful effects of MFs and as such have developed techniques for generating and manipulation of artificial exogenous MFs that mimics these daily exposures as reported by Zanella^35^. Interestingly, a study had concluded that optimal MF exposure for a definite short period of time could surprisingly and significantly promotes cell growth. Many studies have since then, been conducted and have reported^37^ favorable effects of MF exposure such as accelerated healing of bone fractures and halting osteoporosis^42–44^. Accordingly, our study aimed to investigate the potential impact of induced static magnetic field (SMF) on the proliferative tendencies of human umbilical cord-derived mesenchymal stem cells using SmCo_5_. SmCo_5_ whose magnet strength is second only to NdFeB magnet was chosen as a magnetic source in this experiment because it possesses a strong permanent moment magnetization and stable against the influence of demagnetization. It is also suitable for relatively high temperature experiment and rust resistant so no surface treatment such as coating or sealing is needed^45,46^. In this study, a controllable model of SmCo_5_ was designed as the source of MF, and provides a simulation of those long-term effects within a relatively short time. The objective of the present study is to find the optimum culture system by investigating the effects of exposure of SMF SmCo_5_ on the proliferation, growth rate and gene expression of hUC-MSCs with the aim of identifying MF exposure as an alternative technique for increasing the proliferative capacity of MSCs without altering the properties of MSC. Towards this, we provide an essential basis prior to any future SMF SmCo_5_ in vitro experiments.

## Results

### The MF exposed hUC-MSCs maintained morphological integrity

**Figure 1:**
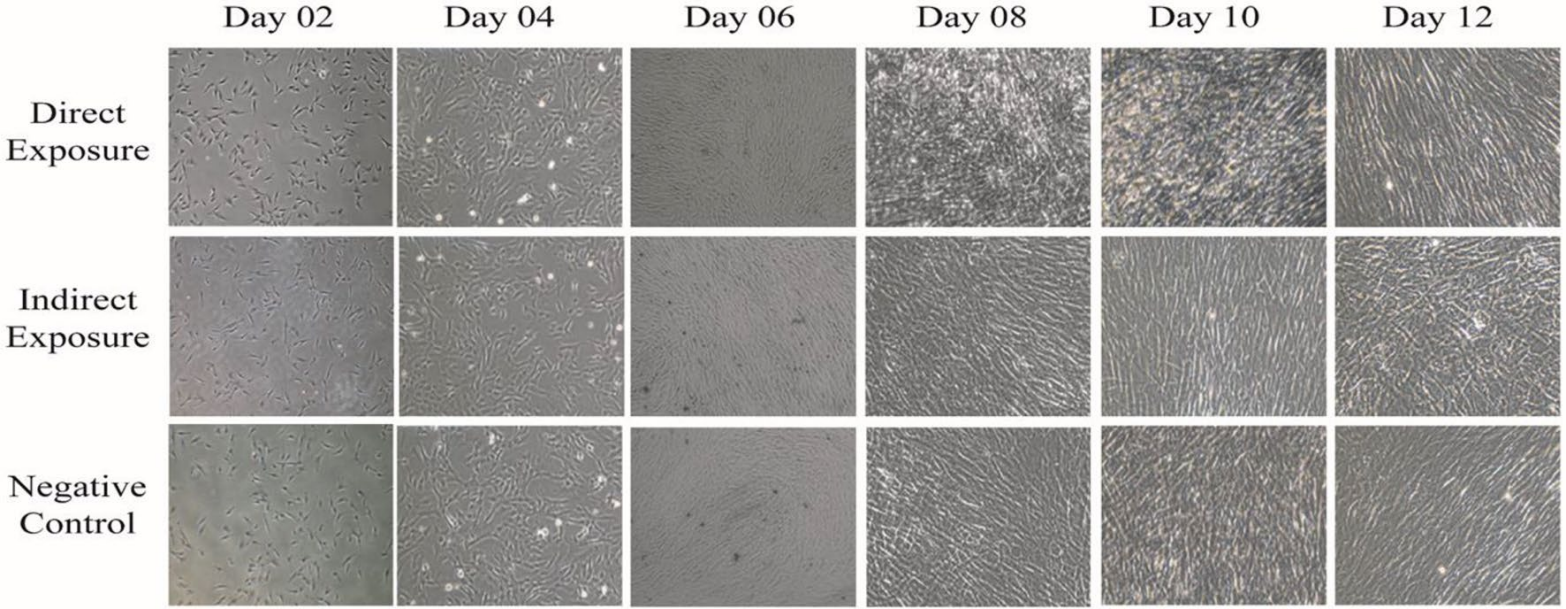
Morphological observations of the hUC-MSCs. Observation of cell morphology of the passage 3 hUC-MSCs in the DE, IE and NC groups. All cells exhibited the characteristic MSC fibroblast-like spindle shape with the DE group achieving 100% confluence at day 8 while the IE group with the least confluency even after day 10. The NC group achieved 100% confluence at day 10. All photo micrographs were taken using CKX41 Inverted Microscope (Olympus, Japan) with 100X magnification.

The observation of the cell morphology was recorded on passage 3 hUC-MSCs cultured from day 0 to day 10 as presented in Fig. 1. For both test groups (DE and IE) as well as the control (NC) group, adherent cells were observed at day 2 with the cells maintaining the elongated fibroblast-like spindle-shape, a characteristic of healthy MSCs. Although the spindle shaped morphology was retained in both groups, all through until day 10, there were noticeable differences in time they required to attain 100% confluence. While the DE group attained 100% confluence at day 8 of culture, cell confluence in the IE and NC groups was approximately at 80%. The confluence however remained unchanged in the IE group but markedly improved in the NC group after culturing to day 10 (Fig. 1).

### The MF exposure affected the growth kinetics and population doubling time of hUC-MSCs

**Figure 2:**
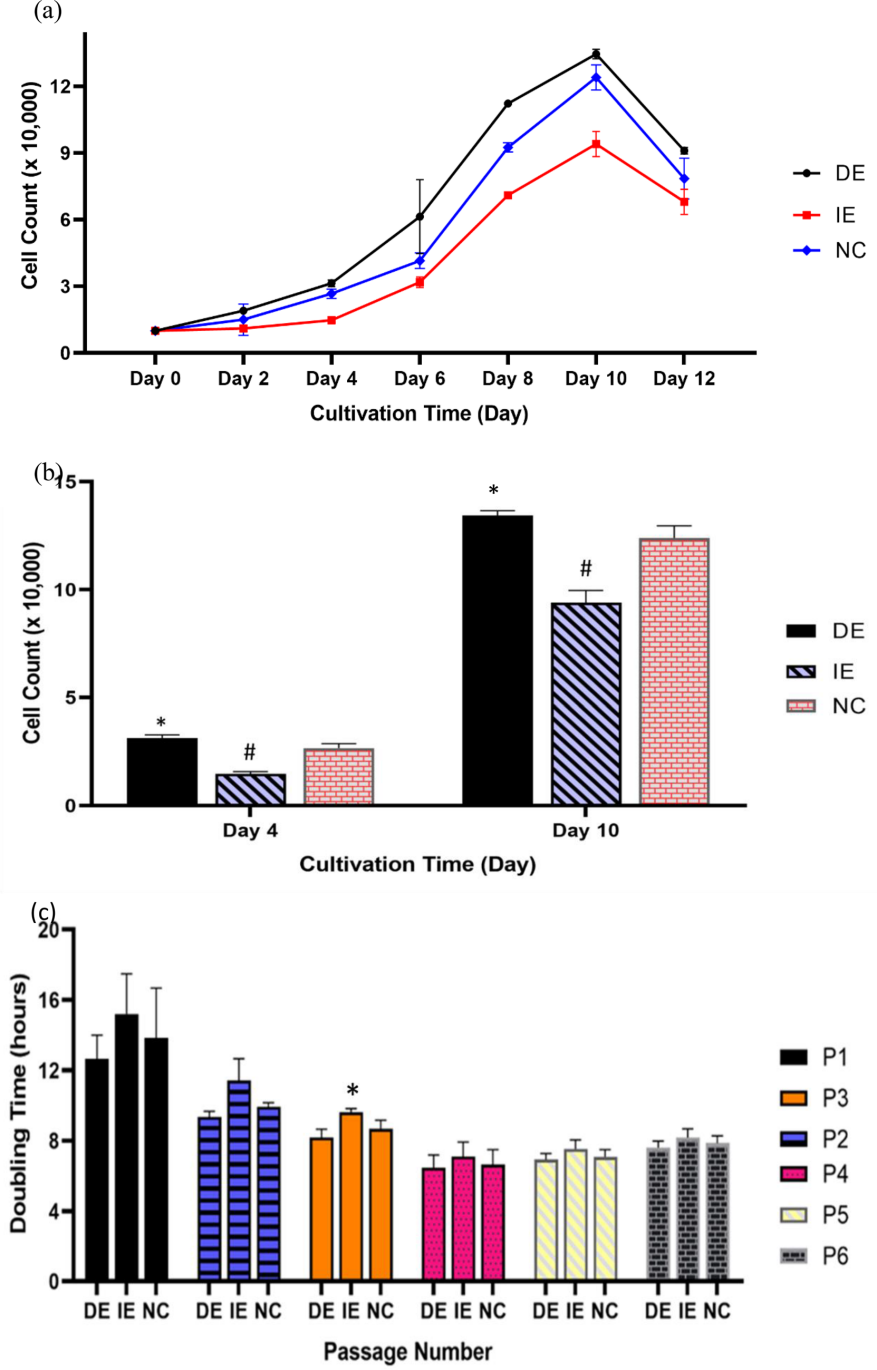
Growth kinetics of the hUC-MSCs showing the effect of direct and indirect MF exposure of the dead cells. (**a**) Similar growth pattern starting with the lag phase, followed by the phase of exponential growth at day 4–10 and terminated of stationary phase was exhibited by cells in direct and control groups. (**b**) Comparisons of the cell count at day 4 (Duration of lag phase) and day 10 (Exponential phase peak) between DE, IE and NC groups.* shows statistical significant increase in compared to the control (NC) while # shows statistical significant decrease compared to the control (NC) (*p* < 0.05). (**c**) Population doubling time (PDT) of the MF exposed (DE and IE) as well as the control (NC) hUC-MSCs from passage 1 to passage 6. The PDT was decreased as the cell expanded to passage 4 after which it plateaued up until 6th passage. * shows statistical significant increase in cell count compared to the control (NC). There no significant difference however, between the PDT of cells in the DE group and those of the NC group (*p* < 0.05).

The results obtained from the growth kinetics showed that the cells in the test and control groups exhibited similar growth pattern which started with the lag phase, followed by the phase of exponential growth, and terminated at the stationary phase Fig. 2a. Although the lag phase lasted till day 4 and the exponential phase peaked at day 10 in the DE, IE and NC groups, there was significant increase in the cell count in the DE group (3.13 × 10^4^ ± 0.11) comparison to the NC group (2.66 × 10^4^ ± 0.21 cells). A significant decrease in cell count was observed in the IE group (1.47 × 10^4^ ± 0.15 cells) in compared to the NC. However, at day 10, where the exponential phase peaked, there was no significant difference between the DE and NC group whereas a significant drop in cell count was observed in the IE group (i.e. IE [9.42 × 10^4^ ± 0.56 cells] against NC [12.4 × 10^4^ ± 0.55 cells]) as shown in Fig. 2b. It was also observed that on reaching the peak of the exponential phase, cells in both tests groups as well as the control, underwent a steep decline in growth (Fig. 2a) rather than the plateau phase, as expected in the stationary phase of growth kinetics. In the population doubling time (PDT) analysis conducted on the hUC-MSCs from passage 1 to passage 6, there was a decrease in the PDT as the cell expanded to passage 4 after which it plateaued up until passage 6. Interestingly, the PDT of cells in IE group was higher relative to that of the DE and NC groups throughout passage 1 to 6 with statistically significant increase (*p* < 0.05) in IE group compared to the NC recorded at passage 3. There is no significant difference however, between the PDT of cells in the DE group and those of the NC group (Fig. 2c).

### The effect of MF exposure on expression of cell surface markers in hUC-MSCs

**Figure 3:**
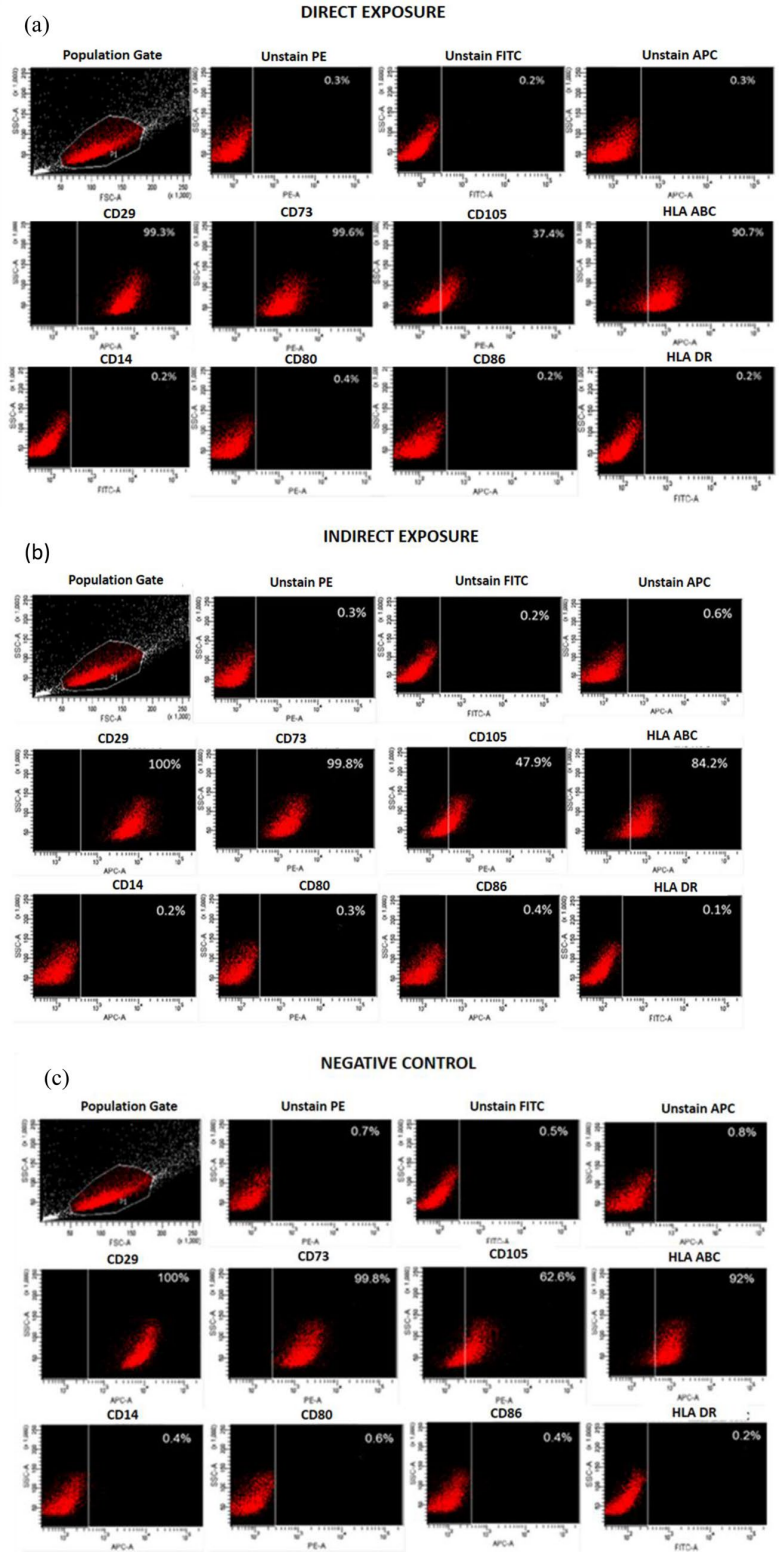

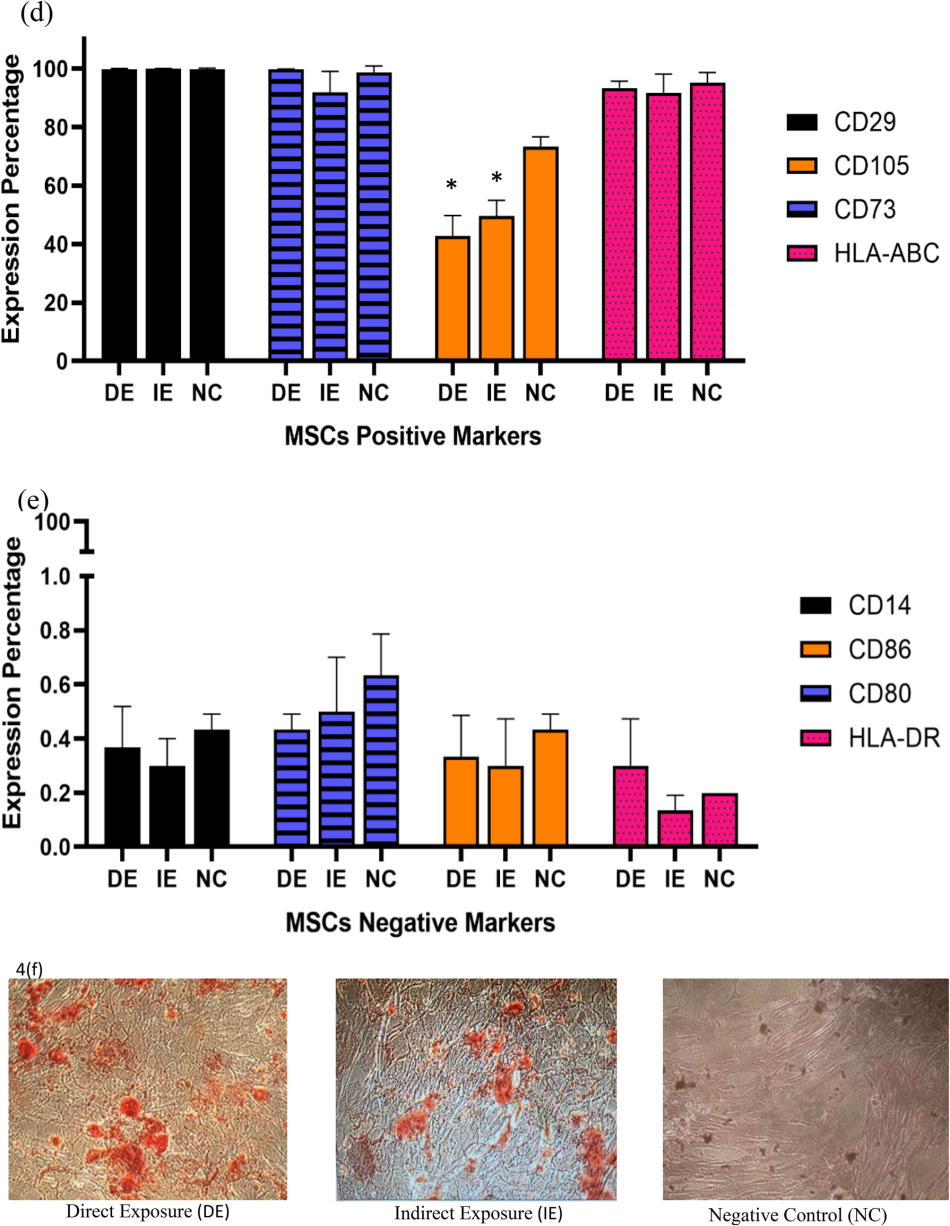
(**a**) Control panel and cell surface markers expression on the directly exposed (DE) hUC-MSCs. The cells exhibited more than 90% positive expression for CD29, CD73 and HLA-ABC with lesser percentage positive for CD105 i.e. only 37.4%, while less than 1% expression for CD14, CD80 and CD86 and HLA-DR was observed. (**b**): Control panel and cell surface markers expression on the indirectly exposed (IE) hUC-MSCs. The cells exhibited more than 90% positive expression for CD29, CD73 and HLA-ABC with lesser percentage positive for CD105 i.e. only 47.9%, while less than 1% expression for CD14, CD80 and CD86 and HLA-DR was observed. (**c**) Control panel and cell surface markers expression on the control (NC) hUC-MSCs. The cells exhibited more than 90% positive expression for CD29, CD73 and HLA-ABC with lesser percentage positive for CD105 i.e. only 62.6%, while less than 1% expression for CD14, CD80 and CD86 and HLA-DR was observed. (**d**): The result shows the expression of the cell surface marker proteins CD29, CD73, and HLA-ABC are similar between DE and IE compare to NC, while, endoglin (CD105) is significantly declined in DE and IE groups compare to NC group. * shows statistically significant group in compare to negative control. (**e**) The result shows the similar expression pattern hematologic and immunologic markers in MSCs without any significant differences between groups. (**f**) Differentiation of hUC-MSCs into osteocytes. MSCs were cultivated to confluence and stimulated to differentiate into osteocytes using appropriate medium for a total of 21 days. According to a cytochemical staining experiment, the hUC-MSCs treated with both test groups (DE, IE) were able to grow into osteogenic cells. Calcium deposition stained with Alizarin Red S revealed osteogenic differentiation. All photo micrographs were taken using CKX41 Inverted Microscope (Olympus, Japan) with 100X magnification.

The characterization of the hUC-MSCs which was performed by evaluating expression of cell surface markers following the exposure to MF indicated that with the exception of CD105, the MF does not completely alter the immunophenotypic integrity of the cells even after 72 h of culture incubation. The results revealed that the cells in all three groups exhibited negative expression for immunological and haematological markers i. CD14, CD80, CD86 and HLA DR, DP, DQ coupled with a positive expression for MSC markers ii. CD29, CD73, CD105 and HLA-ABC (Fig. 3a–e) The antibodies were purchased from Benton Dickinson except CD105 which was purchased from R&D system (Table 1). Additionally, there was no significant difference in the expression levels of the cell surface markers between the DE and IE group compared to the NC group. As presented in Fig. 3a, more than 90% of cells in the DE group have positive expression for CD29, CD73 and HLA-ABC with lesser percentage positive for CD105 (37.4%). A similar trend was observed in the IE group (Fig. 3b) showed > 90% positive expression for CD29 and CD73 with the exception of HLA-ABC and CD105 with 84.2% and 47.9% positive expression respectively. Finally, more than 90% of cells in the NC group (Fig. 3c) have expressed CD29, CD73 and HLA-ABC except CD105 where the percentage is 62.6%. Cells in all three groups had less than 1% expression for CD14, CD80 and CD86 and HLA-DR. Since hUC-MSCs have been positive for MSC expression even after being exposed to SMF, it can be concluded that SMF SmCo_5_ does not contribute to any significant effect or changed immunophenotyping hUC-MSCs even after 72 h culturing in MF environment. Although there was a significant reduction in the expression of CD105, MF did not lead to any significant different in the surface phenotype of MSCs upon treatment (Fig. 3d,e).

**Table 1:** Mouse raised against human MAb used in flow cytometry analsysis.

Antibody	Predominant reactivity	Clone/source
Anti-huCD29-APC	Thrombocyes, monocytes & lymphocytes	MAR4/BD
Anti-huCD73-PE	T,B,DC, endhothelial & stem cells	AD2/BD
Anti-huCD105-PE	Endoglin neuronal axons	166707/R&D system
Anti-huHLA-ABC-APC	Nucleated cells in the body	EMR8-5/BD
Anti-huCD14-FITC	Monocytes	M5E2/BD
Anti-huCD80-PE	Activated B Cells & DC marophages	1.307.4/BD
Anti-huCD86-APC	Activated B Cells & monocytes	2331(FUN-1)/BD
HLA-DR,DP,DQ-FITC	MHC-11 expression cells	TU39/BD

The antibodies were purchased from Benton Dickinson except CD105 which was purchased from R&D System.

*hu* human, *APC* allophyceoerythrin, *DC* dendritic cells, *FITC* fliorescein isothiocynate, *PE* phycoerythrin.

### hUC-MSCs treated with MF showed osteogenic differentiation capacity

In the preliminary investigation of the differentiation study, the DE and IE groups hUC-MSCs were cultivated to confluence and subjected to osteogenic differentiation. The cells were incubated in an osteogenic induction medium for 21 days using a commercially available MSC osteogenic differentiation assay kit. The hUC-MSCs from both test groups were able to undergo osteogenic differentiation and expressed osteogenic markers that were visualised through cytochemical staining. The calcium deposition as a sign of osteogenic differentiation revealed a vivid red colour staining when the cells were grown in the osteogenic inductive media and stained with Alizarin Red S (Fig. 3f)).

### Cell cycle analysis

**Figure 4:**
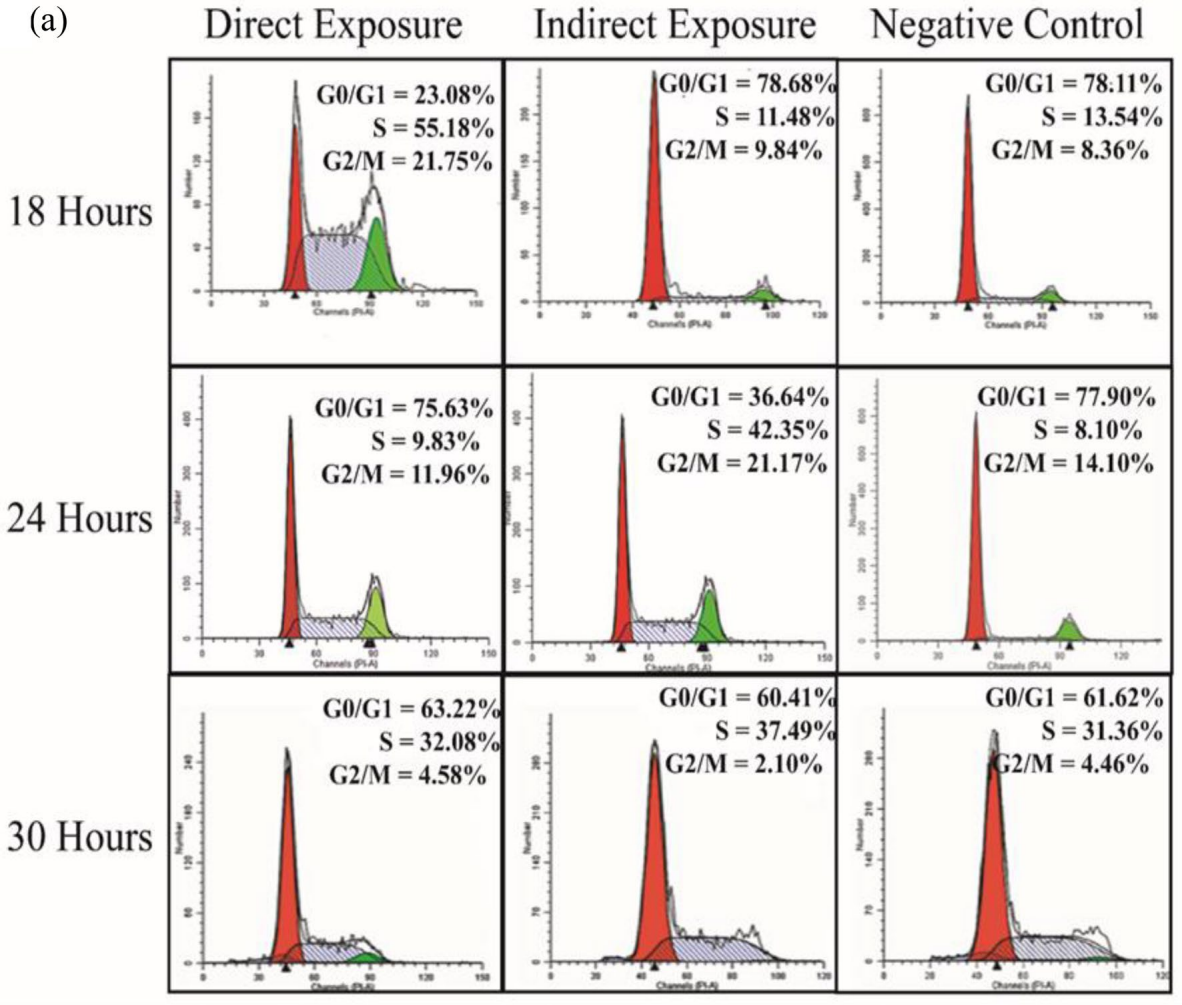

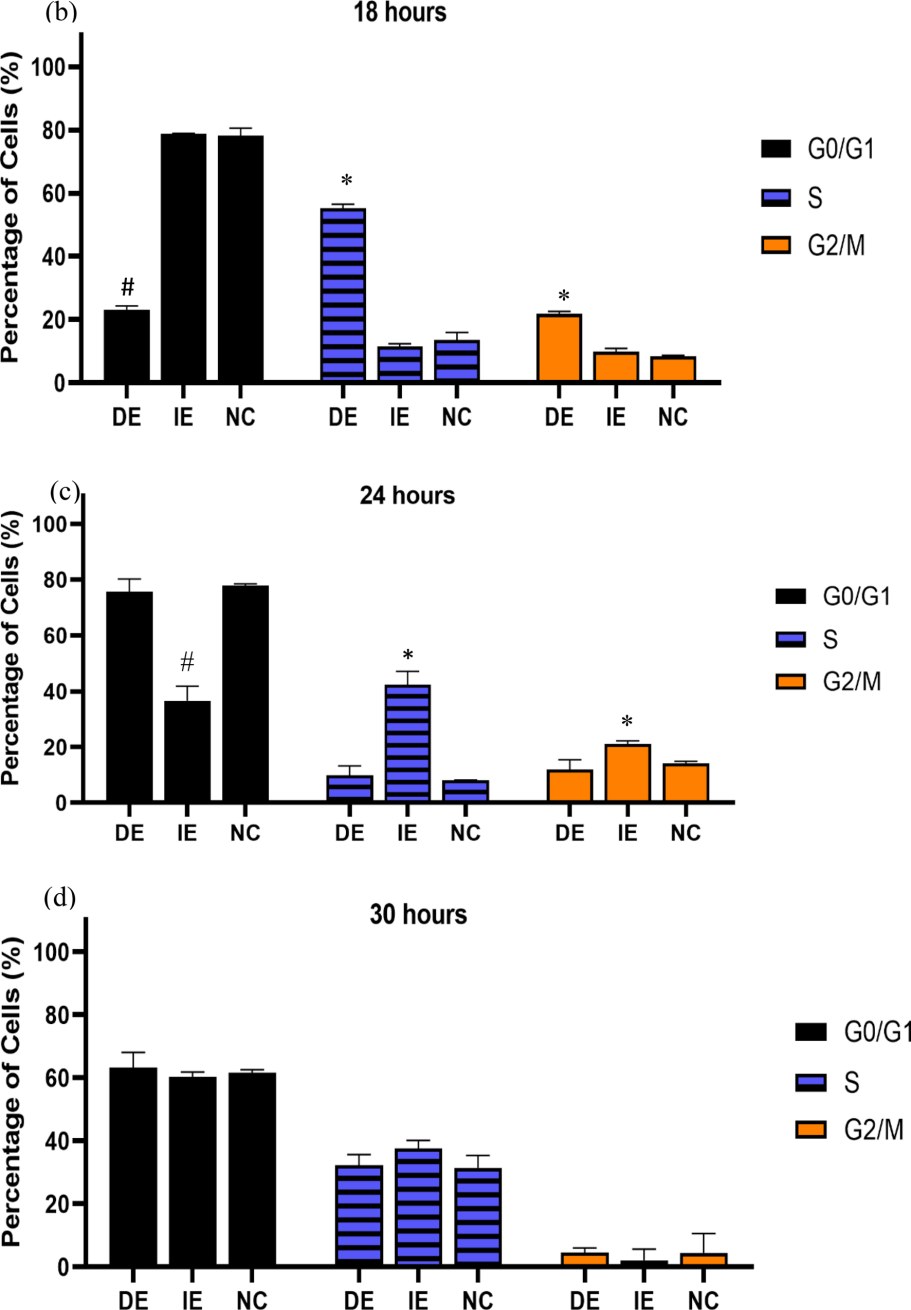
(**a**) DNA distribution showing cell cycle analysis of the MF exposed (DE and IE) as well as the control (NC) hUC-MSCs after 18, 24 and 30 h of culture. The cells in the DE were the first to progress into the cell cycle (as early as 18 h) as the G0/G1 phase markedly decreased while the S- and G2/M-phase increased. The trend was followed by the IE group at 24 h while the NC group lagged till 30 h. Graphical comparison between the cell cycle phases of the DE, IE and NC groups. (**b**) The picture shows the decreased G0/G1 phase and increased S- and G2/M phases in the DE group relative to the NC group after 18 h of culture. (**c**) Shows the decreased G0/G1 phase and increased S- and G2/M phases in the IE group relative to the NC group after 24 h of culture, while (**d**) shows that there was no significant difference in cell cycle phases between the exposed and control groups. * Shows statistically significant increase in percentage of cells compared to the control (NC) while # shows statistical significant decrease in percentage of cells compared to the control (NC) (*p* < 0.05).

Since SMF SmCo_5_ could enhanced the proliferation at exponential phase further experiments were performed to investigate the DNA content of hUC-MSCs via cell cycle. Shown in Fig. 4a, our findings showed that after 18 h, the higher number of cells treated with MF showed a higher PI signal intensity observed after in the S and G_2_/M phase (55.18% and 21.75% respectively) in relative to IE and NC groups, suggesting that the DE group were committed into the S and G_2_/M phase. Finally, factors such as the MF strength of 21.6 mT and exposed periods of MF ranging from 18 to 30 h to hUC-MSCs did not seem to play significant role in the cell cycle for NC. Therefore, this finding suggest the DE SMF treated hUC-MSCs is an optimum condition that allow hUC-MSCs to progress into cell cycle (as early as 18 h). The comparison between 18, 24 and 30 h can be seen at Fig. 4b–d.

### Reverse transcription-polymerase chain reaction (RT-PCR)

**Figure 5:**
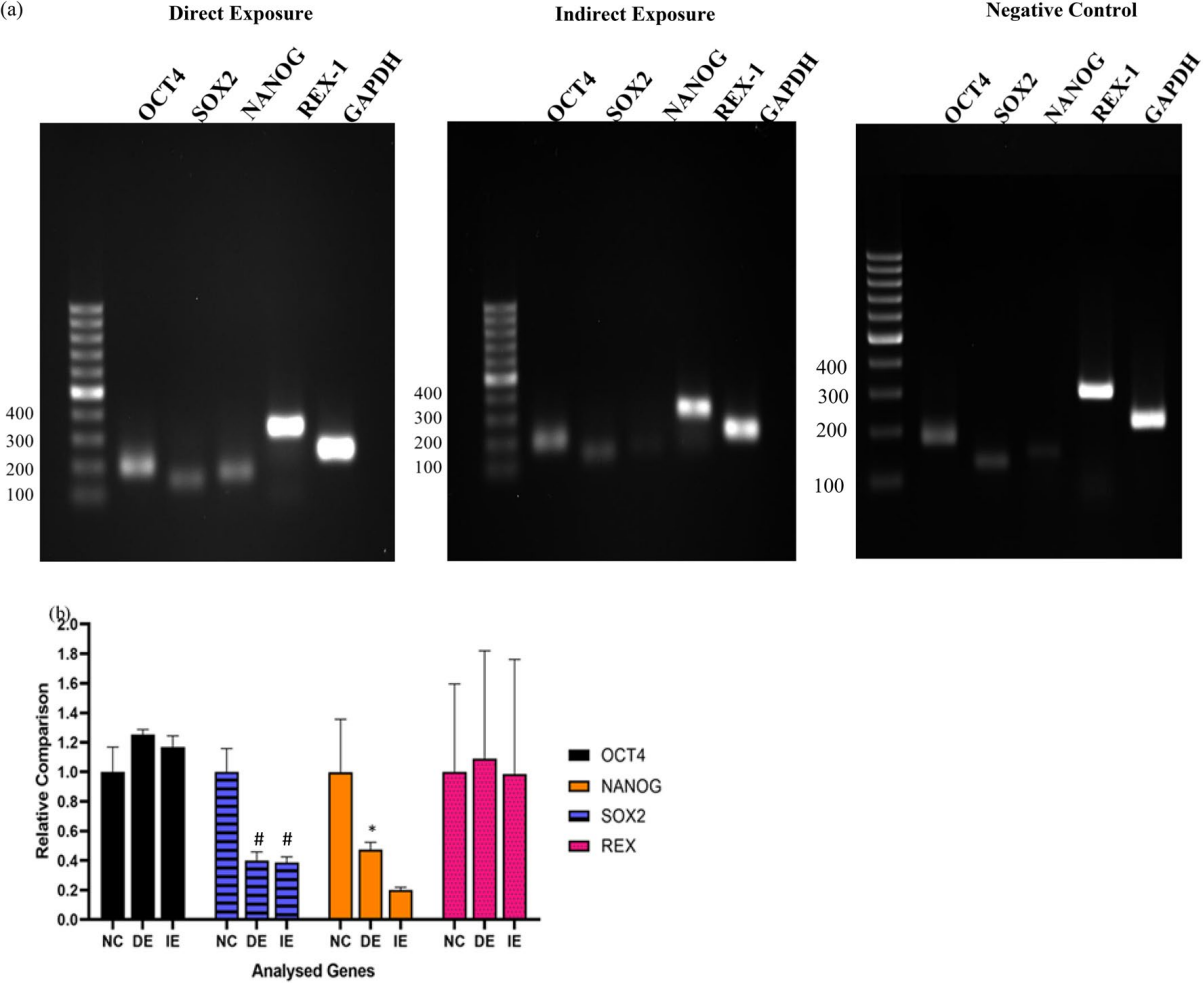
Gene expression of pluripotent-associated markers (*OCT4, SOX2, NANOG* and *REX-1*) in MF exposed hUC-MSCs. (**a**) The RT-PCR product on agarose gel-electrophoresis performed to determine the expression intensity of the markers mainly expressed in undifferentiated MSCs. (**b**) Fold change showing the upregulation of *OCT4* in both the DE and IE groups with expression levels higher in the DE group relative to the IE group. The *SOX2* gene was significantly downregulated in the DE and IE groups, while *NANOG* was significantly upregulated in DE (2.73-fold change) relative to IE groups.

Reverse Transcriptase Polymerase Reaction (RT-PCR) product agarose gel electrophoresis was performed to determine the expression of *OCT4, SOX2, NANOG* and *REX-1* genes which represent an induced stemness markers and mainly expressed in undifferentiated cells of hUC-MSCs. Overall, the treated samples (DE) exhibited a higher expression of these genes in compared to the controls (IE and NC) Fig. 5a. From Fig. 5b, showed that the DE group led to significant increase in the expression of *NANOG* (2.73 folds), relative to IE. There is no significant difference of *OCT4, SOX2* and *REX1* expression was observed between DE and IE groups, although MF exposure to DE group seemed to have a slight increase expression of *OCT4* in compared to IE groups. These results showed that exposure to MF does not reduce the expression of these marker genes but, instead, might be able to maintain the stemness properties of hUC-MSCs (Supplementary File).

## Discussion

In this study, the results observed are indicative of the MF’s potentials in enhancement of the in vitro proliferation of hUC-MSCs. The effects of MF were first observed on the morphological properties of the hUC-MSCs. Although the investigation showed no visible morphological differences were observed between the DE, IE and control (NC) groups, there was noticeable improvement in the speed at which confluence is achieved. In addition, the MF treated cells showed similar morphology to untreated cells (NC), as they maintained the typical appearance and morphology of healthy hUC-MSCs (fibroblast-like and spindle-like shaped) appearance which is in agreement with the studies reported by Marędziak et al.^47^, and Du et al.^48^. This, therefore, suggests that direct exposure to MF may provide a potent method for increasing propagation speed whilst maintaining a normal morphological structures. The positive outcome of growth kinetics time analysis further support the inference made from the morphological observations. The DE cells exhibited a higher cell count at day 4, relative to IE and NC, where the exponential phase initiated, suggesting that the MF exposure remarkably improves the growth kinetics of hUC-MSCs and hence, aids timely with the in vitro propagation. Our observation in this aspect were in contrast to 3 other studies. First, Wiskirchen et al.^49^ revealed that proliferation kinetics were not altered by exposure to MFs and repetitive exposure to a static magnetic field (SMF) of 0.2, 1.0 and 1.5 T exerted no effects on proliferation of human fetal lung fibroblast (HFLF) cells after 21 days. Similarly, contrasting findings have been reported by Miyakoshi^50^ in which, there was no alteration to the proliferation kinetic after cells exposed to the MF. Thirdly, Sun et al., showed that the kinetic analysis of Bone Marrow Mesenchymal Stem Cells (BMMSCs) during the exponential growth phase was not significantly affected by pulsed electromagnetic field (PEMF)^43^. In contrast to their findings, our study has successfully altered growth kinetics, mainly perhaps contributed by the method of magnetic exposure as well as sources of cells used for magnetic field exposures. In addition to the aforementioned observation, the population doubling time decreased passage number increase in both test and control groups. However, shorter doubling time was observed in the DE in compared to the IE and NC groups. This observation is in agreement with the observation by Marędziak et al.^51^, where they reported the highest proliferation rate was observed in human adipose-derived mesenchymal stromal stem cells (hASCs) cultured in the presence of MF conditions and multiplied with the shortest PDT. In contrast, Sadri et al.^52^ reported treated hUC-MSCs with SMF at 18 mT and discovered that the SMF caused the PDT to become longer in comparison with the control group. Here, we have successfully showed that a direct exposure of MF to the hUC-MSC at 21.6 mT SMF is an optimum condition to increase cell proliferation with the shortest PDT.

The characterization of the hUC-MScs upon exposure to MF was further conducted by analysing the cell surface markers have demonstrated that the CD markers of the DE and IE groups were not different compared to the control NC group. The results showed that cells exhibit mesenchymal stem cell-like phenotypes even after 72 h of culturing in MF-exposed environment. The MSCs constitute a heterogeneous population of cells, in terms of their morphology, physiology and expression of surface antigens. Until now, there are no single specific cell surface markers identified and generalized for MSCs. In this study we have shown that the hUC-MSCs isolated using flow cytometry was homogenous without any contamination from hematopoietic stem cells and their progenitors. This was confirmed by our immunofluorescence experimental data which represented the positive markers CD29, CD73, CD105 and HLA-ABC, and against lack expression of negative markers CD14 and HLA-DR and the co-stimulatory markers CD80, CD86 in the cultures. It is important to note however, that the expression levels for CD105 declined significantly in the exposed groups i.e. DE and IE. The reduced expression of CD105 in DE and IE in relative to the NC suggest a potential alteration to their cell fate. Whether the observed decrease could be associated with a suppressed or enhanced biological function, this warrants further investigation. Apart from CD105, there was no significant difference in expression levels of the cell surface markers between the exposed and controls. Similar results have been obtained by Sun et al.^43^ as they reported that MF exposure does not significantly affect the observed surface phenotype morphology and multi-lineage differentiation potential for the BM-MSCs. Since the hUC-MSCs have been positive for MSCs expression (surface adherence and cell surface expressions) even after being exposed to MF, it can be concluded that the MF does not contribute to any significant effect or changed immunophenotype hUC-MSCs by the MF even after 72 h culturing in MF environment.

When cultured in an osteogenic induction medium, the culture-generated hUC-MSCs were differentiated into osteocytes based on the cytochemical staining. Specifically, the innate fibroblast-like morphology of MSCs has changed into cuboidal-like shapes during induced osteogenesis. When these cells were stained with Alizarin red, they appeared to be brick-red in colour due to cell aggregation and nodules formation. A particular region of the staining pigment appeared denser and is believed to be calcium deposition, a common osteogenesis indicator. The results from the current study are similar to the studies conducted by^53–55^. However, further characterization of osteogenesis should be performed to validate this observation thoroughly. For instance, the calcium content of these cells can be assessed further via a colourimetric assay, and RT-qPCR can be utilized to identify the presence of osteocytes markers. Additionally, western blot analysis can evaluate the presence of proteins related to osteogenesis. Our preliminary findings here support the notion that SMF SmCo5 does not affect the multipotent differentiation potential of hUC-MSCs, particularly osteogenic potential.

Proliferation assays using techniques such as MTT and cell cycle, have shown increased proliferation of MSC following exposure to MF^51,52,56^. Our result showed that the cells in the DE group were committed into the cell cycle as early as 18 h of cultured when compared with the IE and NC groups. Over time, hUC-MSC may experience cellular senescence where more cells are arrested in G_0_/G_1_ phase^57^. Aging of cells is closely related to the telomerase activity and telomere length. Although the telomere length and the telomerase activity were not measured in the presence study, the promotion of cell cycle progression and diminution of apoptosis activity as indicated by cell cycle may indirectly represent telomerase activity. This suggests that MF intensified may induce hUC-MSCs survival or MF act as anti- apoptotic agent for the cell cycle mechanism from 6 to 48 h. The cells may possibly exit the resting state and continue proliferating; and thus, the aging process may be prevented by MF-exposure. To confirm this, measurement of apoptosis activity such as caspase assays should be conducted for further investigation. The results obtained showed the MF increases the number of cells at detected S and G_2_/M phase as observed in the DE group.

Our RT-PCR gene expression analysis revealed that *NANOG* expression increased 2.73-fold in DE relative to IE, at passage 3. This is in line with the findings by Rinaldi et al.^58^ where exposure to Radio Electric Asymmetric Conveyer (REAC) caused 30-fold increase of *NANOG* expression in MSCs, even in cells at passage 30. This consequently induced upregulation of Bmi1 expression that plays a central role in DNA repair and self-renewal of stem cells. As such, MF in the study may facilitate cell proliferation through increased *NANOG* as observed with REAC. The reduced expression of *SOX-2* gene suggests that a more differentiated sub-population of cells were forming in the DE treated cells in compared to the IE and NC. This observation justifies the fact that the cell fate is indeed affected by SMF through some biological phenomenon which warrants further investigations.

The present study revealed that prowess of MF of 21.6 mT intensity in stimulating in vitro proliferation and improving the propagation of hUC-MSCs without affecting its immunophenotypic integrity. However, this prowess can be explored further by analysing the effects on MF exposure on key signalling pathways using global gene evaluation and computational biology tools. From the current studies were geared more towards discovering the fundamental potential of MF exposure in MSCs’ expansion. The increased gene expression of *NANOG* amongst other associated pluripotent markers such as *OCT4, SOX2,* and *REX-1,* may indicate a potential adverse event, as that such co-expression of *NANOG* and *OCT4* in previous literatures had reflected in poor prognosis of several malignancies including lung, glioma, and renal cell carcinomas^59–62^. Therefore, a separate study will be conducted to delve on the matter in a more in-depth manner in the hopes of providing a more concrete conclusion on its safety. Further investigation would also include various genetic and metabolomics studies, investigation on the MF exposures with the stemness and growth of cancer stem cells and the implication on cancer development. Additionally, an important observation made in the present study is the poor performance of indirect exposure of MF towards hUC-MSCs, as evident from the outcomes of the cells IE group throughout the study. Although the IE group was introduced as a technical control, it showed that there is a possibility of an undesirable effect of exposing MSCs to MF indirectly. However, it is recommended that future study be conducted should consider the distance of the magnet source towards the biological properties of MSCs.

## Materials and methods

### Source of static magnetic field and incubator set-up

**Figure 6:**
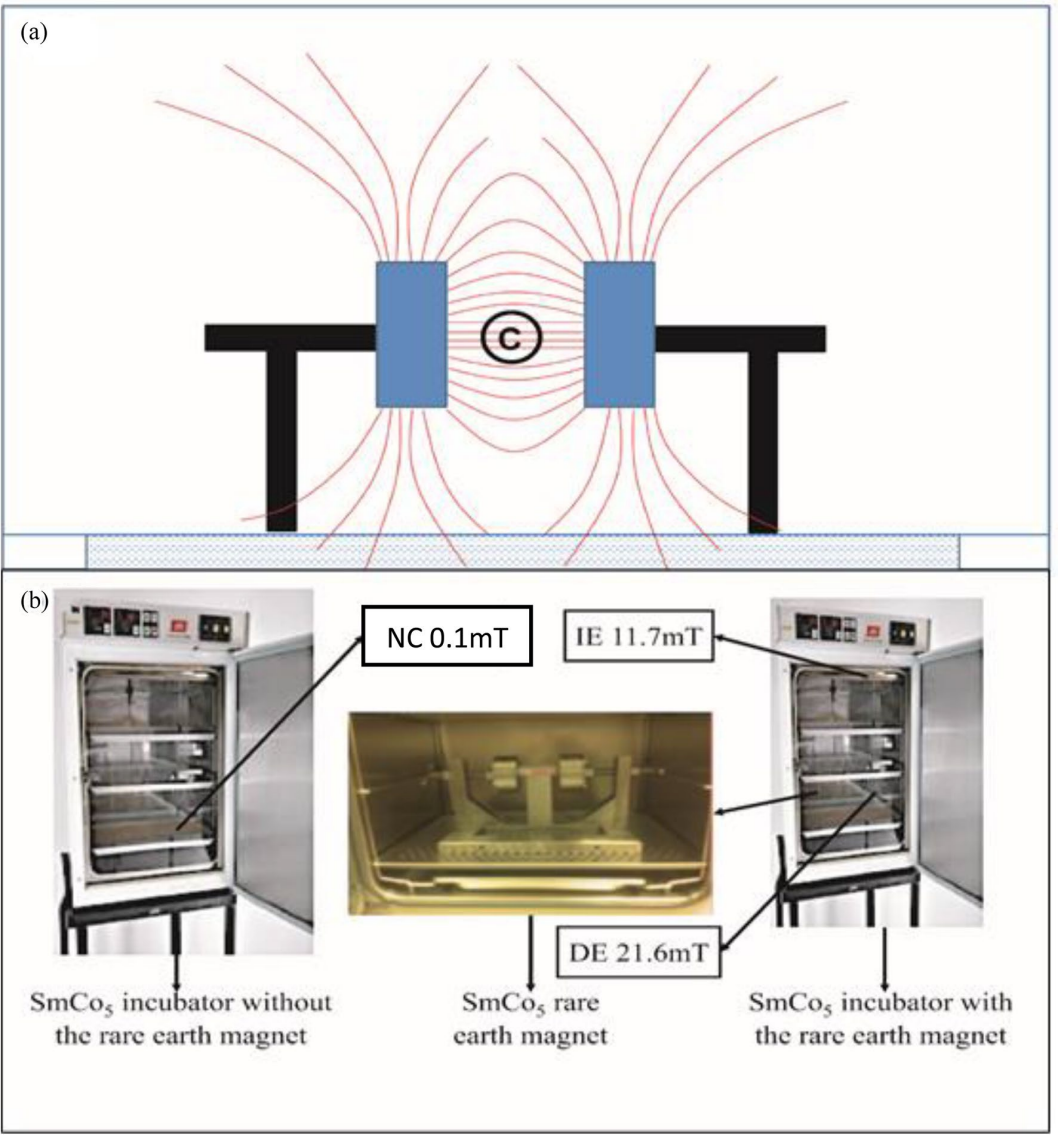
(**a**) The schematic diagram above shows the distribution of MF lines between a pair of rare earth magnet. The centre region between the two magnet cylinders © is where the MF most uniform and intense. (**b**) Setup showing how cells in the direct exposed (DE) and indirect exposed (IE) groups were placed at different locations in the incubator. The DE group and IE group are in the same incubator and the negative control (NC) group in a different incubator.

Samarium-cobalt, a rare earth magnet made of samarium and cobalt alloy that offers large and permanent magnetization was utilized in the study. The magnetic component consisted of two samarium-cobalt magnet cylinders (SmCo_5_ or SmCo Series 1:5). Each magnet cylinder was 4 cm thick with a diameter of 9.5 cm coated with nickel to avoid flaking and to make the surface tougher. The SmCo_5_ magnet cylinders were installed inside the CO_2_ incubator (Galaxy S., USA) with the help of an adjustable positioner with north and south poles facing each other. Since SmCo_5_ magnets are anisotropic, the MF was generated in only one direction. Figure 6a shows the schematic distribution of MF intensity from the surface of the permanent magnet. The highest MF intensity was 21.6 mT, was measured and recorded using Gauss meter (DC Gaussmeter Model GM 1-ST, Type ALPHALAB Inc, USA), placed on top of the cell cultures at the center between the permanent magnets where the MF was thoroughly uniform (Fig. 6b). The incubator has three compartments i.e. the upper, middle and lower compartments with the SmCo_5_ magnet kept in the lower compartment.

### Isolation and culturing hUC-MSCs

Fully characterized hUC-MSCs were obtained from Stem cell & Immunity Research Group, Immunology Laboratory, Department of Pathology, Faculty of Medicine and Health Sciences, University Putra Malaysia. Umbilical cord MSCs were derived from Wharton’s Jelly of the human umbilical cords. The human umbilical cords were obtained from full-term deliveries at the Britannia Women and Child Hospital, Kajang, Selangor, Malaysia, after informed consent was obtained from all subjects and/or their legal guardian(s). The collection and use of human umbilical cord tissues were approved by the Ethical and Research Committee, Faculty of Medicine and Health Sciences, Universiti Putra Malaysia. All methods and procedures of using human cells were performed in accordance with relevant guidelines and regulations, including the Declaration of Helsinki. The cells were cultured in F12 Dulbecco's Modified Eagle Medium (DMEM–F12) composed of Dulbecco GLUTAMAX (Gibco, United Kingdom) supplemented with 10% Fetal Bovine Serum (FBS), 1% Penicillin/Streptomycin, 0.5% Fungizone and 0.1% Gentamycin (Gibco, United Kingdom). The optimised culture medium for hUC-MSC was freshly prepared as described in Tong et al.^63^. Based on the experimental design, the cells were grouped into three, i-the DE group was positioned at the centre between the permanent magnets where the intensity of the MF 21.6 mT as measured by the Gauss meter) was the highest and uniform; ii- the IE group was placed in upper compartment of the same incubator as the DE group, while iii- NC group was placed in a different incubator free from MF exposure. The arrangement of the cells in the incubator was based on the experimental set-up and is presented in Fig. 6b. All culture conditions in the incubators used were maintained at 37 °C and supplied with 5% CO_2_.

### Morphological observation

The hUC-MSCs were seeded in a 60 mm petri dish at 1 × 10^4^ cells and cultured until passage number 6. The morphology of the cells was observed every two days using a phase contrast CKX41 Inverted Microscope (Olympus, Japan). The images obtained for the test (DE and IDE) and the control (NC) groups at passage 3 were recorded.

### Growth kinetics and doubling time analysis

In the growth kinetic experiment, 1 × 10^4^ hUC-MSCs at passage 3 were seeded in 60 mm petri dish. For each group, the cells were seeded in different petri dishes for 6 incubation time points, day 2, 4, 6, 8, 10, and 12. On maturation of each incubation day, the cells were harvested by trypsinization using TrypLE™ Select Enzyme (ThermoFisher Scientific, USA) and were counted by trypan blue exclusion test using Trypan blue solution (Sigma-Aldrich, USA) for. The data was analysed post enumeration. Similarly, for the doubling time analysis**,** 1 × 10^4^ hUC-MSCs at passage 3 were also seeded in 60 mm petri dishes and treated under the same conditions as described above. The cells were harvested by trypsinization using TrypLE™ Select Enzyme (ThermoFisher Scientific, USA) and then were counted manually by hemocytometer under a microscope. The growth rate, which is reciprocal of generation time, is defined as the number of cells doubling per unit of time during a specific time interval. Doubling time was determined by Patterson Formula and expressed as mean doubling time.

### Immunophenotyping

The hUC-MSCs (2 × 10^5^ cells) at passage 3 were cultured in T-25 tissue culture flasks and incubated for 72 h. After approximately 80–90% confluence was achieved, the cells were harvested, counted and 1 × 10^6^ cells were transferred into fluorescence activated cell sorting (FACS) tubes. The cells were then washed in cold 1 × phosphate buffer saline (PBS) and stained with 1.5 μL per 10^5^ cells of panel MSCs anti-human monoclonal antibodies: as stated in Table 1. The stained cells were incubated for at least 15 min at 2–8 °C. After that, the cells were washed and resuspended in 500 μL of PBS and the cell surface markers were detected by acquisition of 10^4^ antibody labelled cells using a BD LSR FORTESSA flow cytometer (BD Bioscience, USA). Unstained and fluorochrome-conjugated non-specific isotype labeled cells were used as a control parallel to all measurements to set negative gating. The data obtained were analysed using Cell Quest Pro software provided by the manufacturer (Becton Dickinson CellQuest software, USA).

### Mesodermal potential analysis: osteogenic differentiation assay

Passage 3 hUC-MSCs were tested for osteogenic differentiation capacity as part of mesodermal potential assessment using StemPro Osteogenesis Differentiation Kit (Gibco, Invitrogen, USA). For the DE group, 10,000 hUC-MSCs cells were seeded in T-25 flasks supplemented with complete MSC media and were incubated at 37 °C and 5% CO_2_ conditions until a monolayer of confluence cells was attained. Upon achieving confluence, the cell culture media was replaced with an osteogenic differentiation medium every 3 days for another 21 days. For the control group, the cells were maintained in complete MSC media only throughout the study. At the end of day 21, cells were harvested and fixed with 70% ethanol for 60 min. This is followed by staining with Alizarin Red solution for 30 min. All cell cultures were observed under a phase-contrast CKX41 inverted microscope (Olympus, Japan), and their images were captured.

### Cell cycle analysis

The hUC-MSCs were seeded in 6 well plates at the number of 5 × 10^4^ cells per well. The cells were grouped into 3 different time points (18, 24, and 30 h) and incubated accordingly. After each incubation period, the cells were harvested, washed in cold PBS and then fixed with 70% ethanol for overnight at − 20 °C. The fixed cells were then washed twice and suspend in 25 µL of 10 mg/mL RNase (Sigma-Aldrich, USA) and incubated with 500 µL staining buffer consisting of 1 mg/mL propidium iodide (PI) stain (Molecular Probe, Invitrogen) incubated at room temperature for 30 min. The DNA distribution of the cell were analysed by acquisition of 10^4^ PI labelled cells using a BD LSR FORTESSA flow cytometer (BD Bioscience, USA). Analysis of the data acquired was performed using ModFit LT Software (Verify Software House, USA).

### Reverse transcriptase-polymerase chain reaction (RT-PCR)

All standard precautions in handling RNA were considered to avoid contaminations. Total RNA from cell pellets was extracted using RNeasy Mini Kit (Qiagen, Germany) according to manufacturer’s protocol. The extracted RNA was eluted to 50 µl with RNase-free water by centrifugation at 13,000 rpm for 1 min. The RNA concentration and purity were measured using Nano-Drop ND-1000 Spectrophotometer (Thermo Fisher Scientific Inc., USA). The RNA (500 ng) was loaded into a well of 1.5% agarose gel (Sigma Sigma-Aldrich, USA) and electrophoresed at 90 V for 60 min. The integrity of purified RNA was assessed by visualization of the 28S and 18S ribosomal RNA bands. The cDNA was generated using QuantiTect Reverse Transcription kit (Qiagen, Germany) according to the manufacturer’s protocol. One microgram (1 µg) of purified RNA was used to generate the cDNA. The synthesized cDNA was stored at − 20 °C and subsequent RT-PCR was performed using a standard 40 cycle protocol. Essentially after an initial denaturation at 94 °C for 10 min, 40 cycles (denaturing at 94 °C for 45 s, annealing at 58 °C for 30 s, extension at 72 °C for 90 s) followed by a final extension at 72 °C for 5 min. RT-PCR amplicons were separated on a 2% agarose gel (SeaKem® LE Agarose, Lonza, Switzerland) and stained with ethidium bromide. The gel was visualized using Syngene Gel Documentation (Syngene, USA). The design of the primers *GADPH, REX 1, SOX2 OCT4* and *NANOG* were adopted from the previous study as presented in Table 2. The band intensity of the genes of interest obtained from the RT-PCR product agarose gel-electrophoresis was quantitated using the Image J™ software, normalized against the reference gene (*GADPH*) and then presented as fold change in reference to the NC group using the formula below.

**Table 2:** Primer sequence of the pluripotent genes and the PCR product size.

Genes	Sequence (5’–3’) Forward and Reverse Primer	Amplicon size (bp)	Annealing temperature (°C)
*GADPH*	TCCCTGAGCTGAACGGGAAG GGAGGAGTGGGTGTCGCTGT	217	58
*OCT4*	GAAGTGAGGGCTCCCATAGC AAGGATGTGGTCCGAGTGTG	180	58
*SOX2*	TTACCTCTTCCCACTCCA GGTAGTGCTGGGACATGTGAA	132	58
*REX-1*	CAGATCCTAAACAGCTCGCAGAAT GCGTACGCAAATTAAAGTCCAGA	306	64
*NANOG*	CTGTGATTTGTGGGCCTGAA TGTTTGCCTTTGGGACTGGT	153	58

$$Fold\,Change = \frac{Band\,intensity\,of\,exposed\,group \left(DE\,orIE\right)-Band\,intensity\,of\,control\,(NC)}{Band\,intensity\,of\,control\,(NC)}$$

### Statistical analysis

The results obtained from treated cells and control cells at different seeding of hUC-MSCs by Student’s t-test using GraphPad Prism (Version 7) and significant levels were set at *p* value < 0.05.

## Supplementary Information

Supplementary Information.

## Abbreviations

%: Percentage
±: Plus minus
°C: Degree Celsius
^3^H-TdR: Tritiated thymidine
BMMSCs: Bone Marrow Mesenchymal Stem Cells
cDNA: Complementary deoxyribonucleic acid
CO_2_: Carbon dioxide
CPM: Count per minute
CD: Cluster of designation
DC: Direct current
DE: Direct exposure
DMEM: Dulbecco’s Modified Eagle Medium
DNA: Deoxyribonucleic acid
EASCs: Equine adult stem cells
ELF: Extremely low frequency
ELF-MF: Extremely low frequency-magnetic field
GADPH: Glyceraldehyde-3-phosphate dehydrogenase
GHz: Gegahertz
FACS: Fluorescence activated cell sorting
FDA: Food and Drug
G0: Quiescence phase
G_1_: Gap phase
G_2_: Second gap
HBS: Human Bovine Serum
HFLF: Human fetal lung fibroblast
HLA: Human leukocyte antigen
hUC-MSCs: Human Umbilical Cord Derived Mesenchymal Stem Cells
Hz: Hertz
ISCT: International Society for Cellular Therapy
IE: Indirect exposure
KV: Kilovolt
M: Mitosis
m: Meter
MF: Magnetic field
mg: Milligram
MHC: Major histocompability complex
MHD: Magneto hydro dynamic
mm: Millimetre
MRI: Magnetic resonance imaging
mT: Militesla
MW/h: Megawatt
NANOG: Pron.nanOg
NC: Negative control
NdFeB: Neodymium ferum boron
ng: Nanogram
OCT4: Octamer binding transcription factor-4
PBS: Phosphate buffer saline
PDT: Population doubling time
PEMF: Pulsed electromagnetic field
PI: Propidium iodide
REAC: Radio electric asymmetric conveyer
RNA: Ribonucleic acid
RNAse: Ribonuclease
RT-PCR: Reverse transcriptase polymerase chain reaction
rpm: Rotation per minute
S: Synthesis phase
S/C: Superconducting
SD: Standard deviation
SmCo5: Samarium Cobalt
SOX2: SRY (sex determining region Y)-box 2
SMES: Superconducting magnetic energy storage
SMF: Static magnetic field
T: Time
Td: Doubling time
T: Tesla
TGF-β: Transforming growth factor beta
TF: Tissue factor
VDT: Video display terminal
µg: Microgram
µCi: Micro Currie
µL: Microliter
µT: Microtesla
REX1: Zinc finger protein 42 homolog
