# Supplementary material for: Magnetic exposure using Samarium Cobalt (SmCO5) increased proliferation and stemness of human Umbilical Cord Mesenchymal Stem Cells (hUC-MSCs)
Source: Sci Rep. 2022 May 26;12:8904. doi: 10.1038/s41598-022-12653-z (PMC9135697; doi:10.1038/s41598-022-12653-z)
Supplement: Supplementary file 1 — Supplementary Information. [file 41598_2022_12653_MOESM1_ESM.docx]

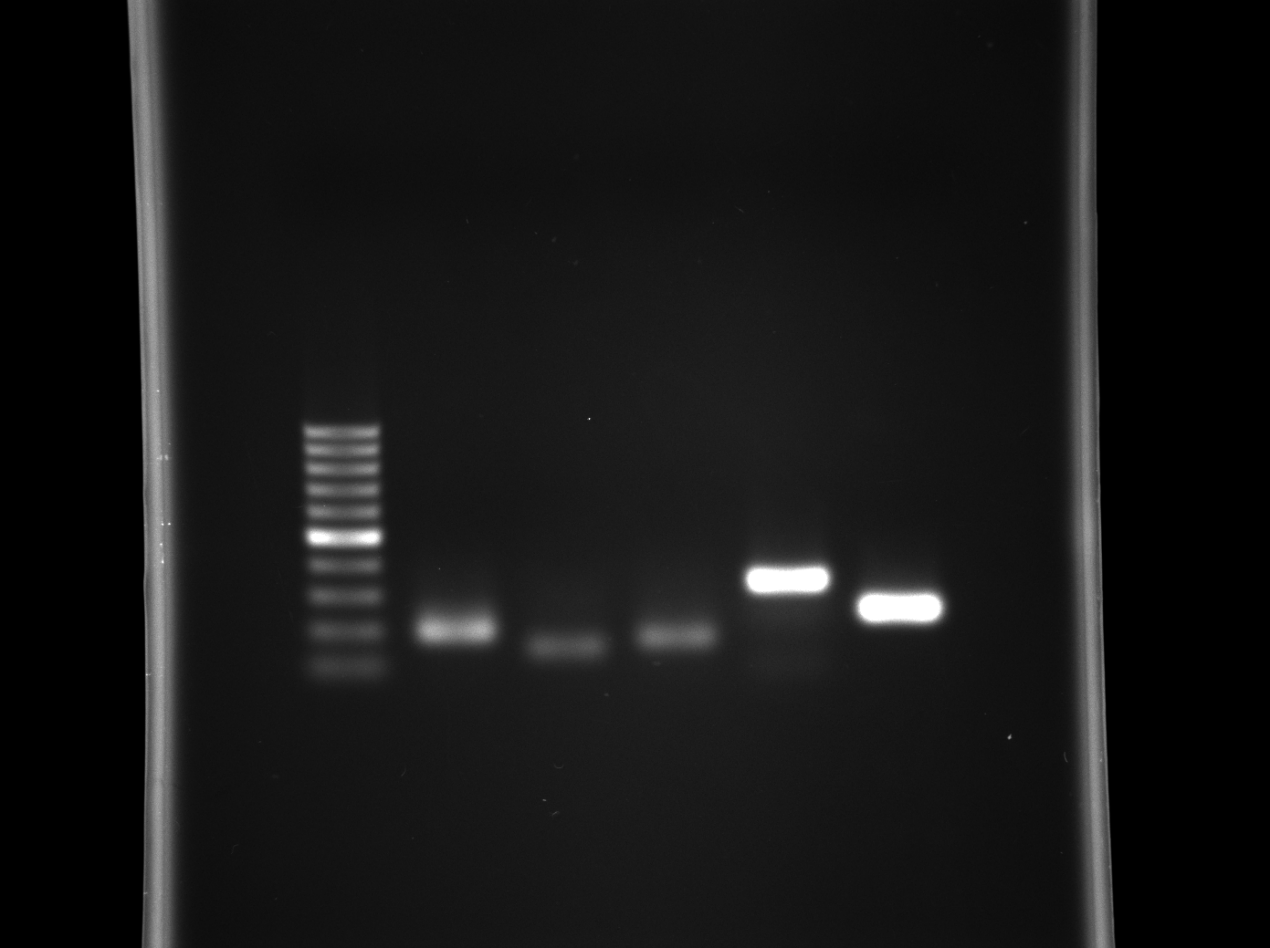


Original picture of Direct Exposure Gel


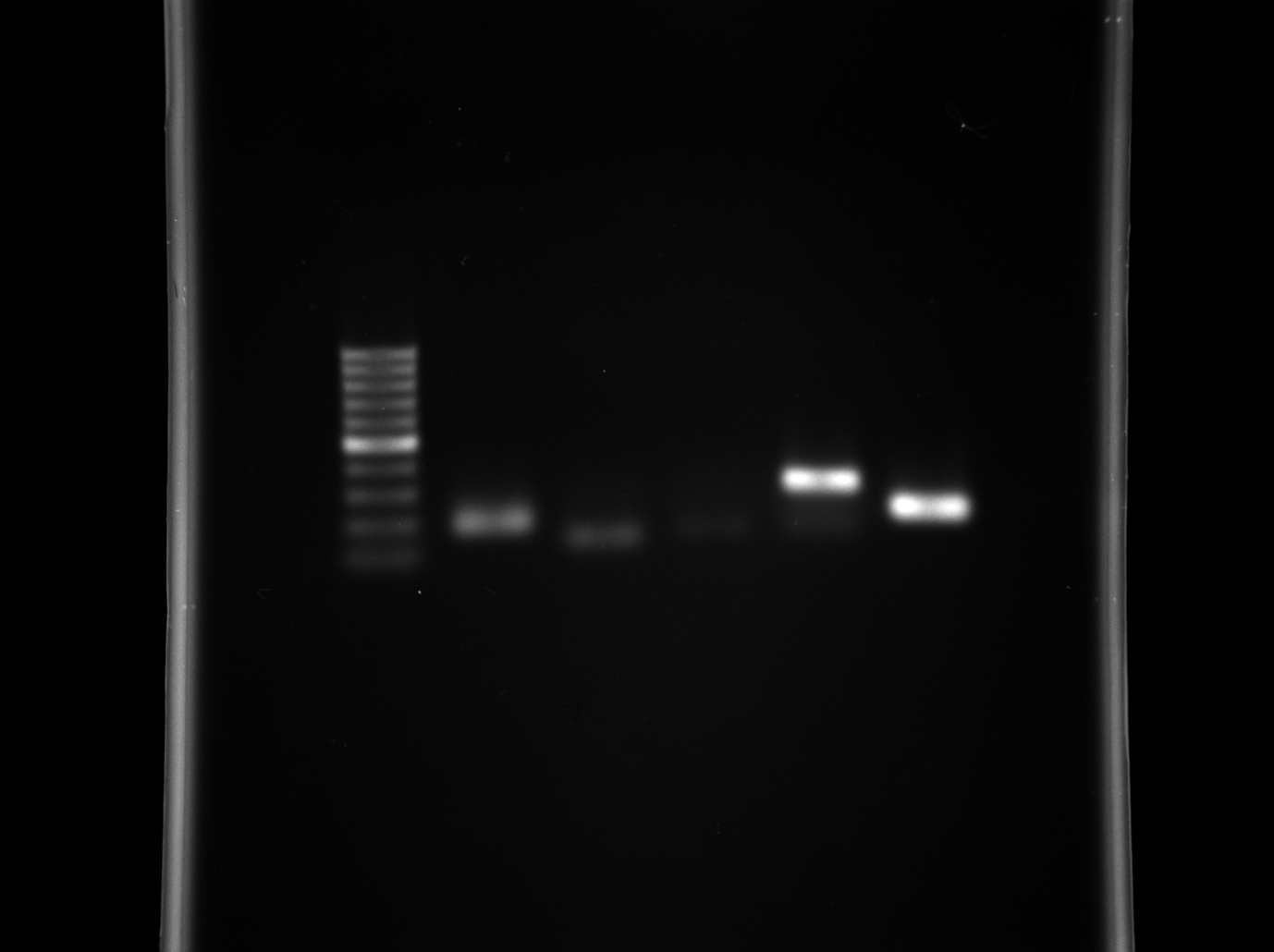


Original picture of Indirect Exposure Gel


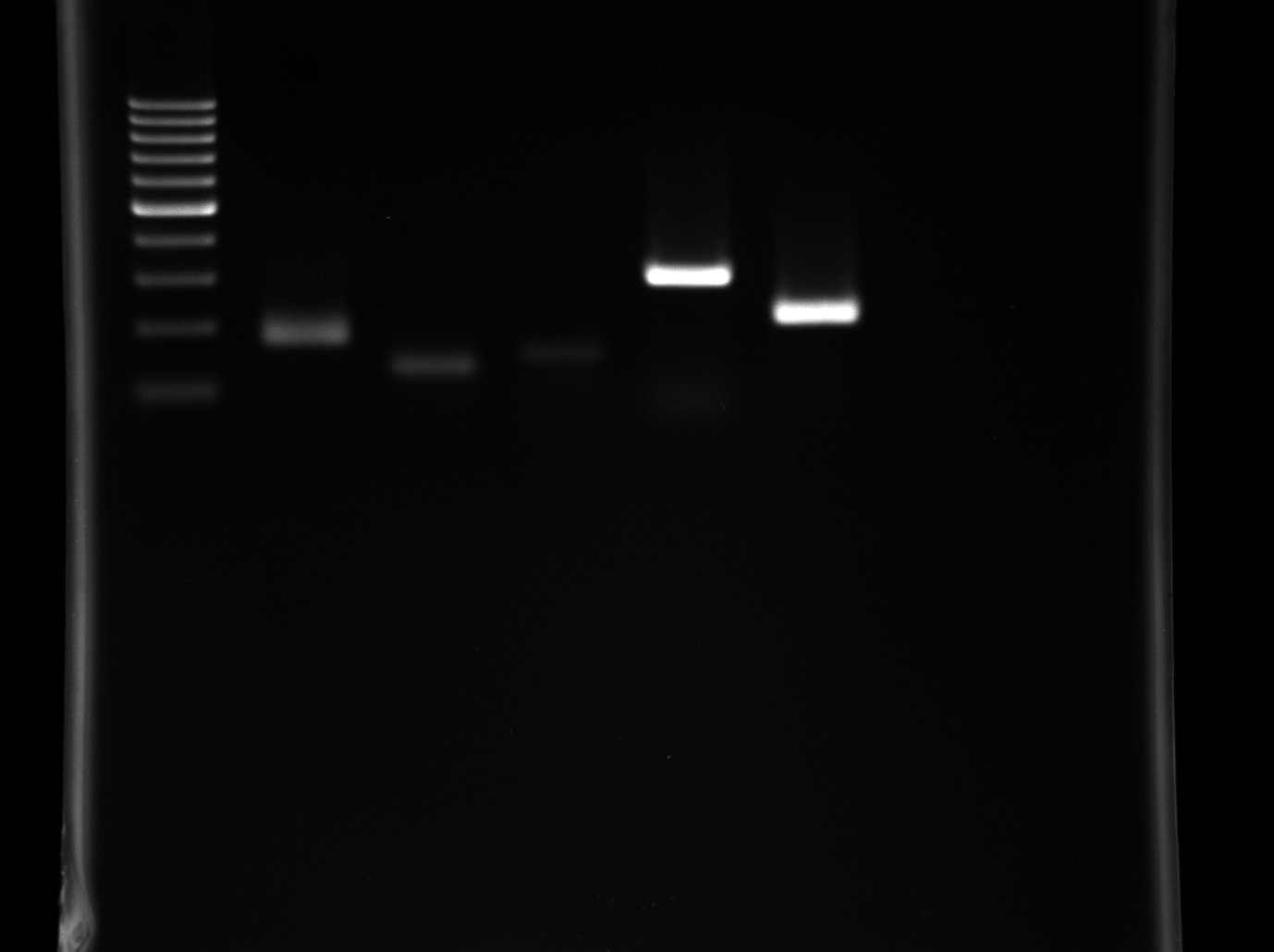


Original picture of Negative Control Gel
